# Water in nanoporous hexagonal boron nitride nanosheets: a first-principles study

**DOI:** 10.3762/bjnano.16.39

**Published:** 2025-04-11

**Authors:** Juliana A Gonçalves, Ronaldo J C Batista, Marcia C Barbosa

**Affiliations:** 1 Departamento de Física, Universidade Federal do Rio Grande do Sul, Av. Paulo Gama, Farroupilha, 90040-060, Porto Alegre - Rio Grande do Sul, Brazil; 2 Departamento de Física, Universidade Federal de Ouro Preto, Campus Morro Campus Morrodo Cruzeiro, 35400-000, Ouro Preto, MG, Brazil

**Keywords:** boron nitride, DFT, nanoporous materials, water

## Abstract

Nanoporous membranes are being explored as efficient materials for water filtration and desalination applications. In this study, we analyzed the behavior of pores within a freestanding hexagonal boron nitride (*h*-BN) monolayer in contact with water molecules. Our investigation revealed that triangular and rhombic pores induce wrinkles in non-deposited *h*-BN monolayers because of the repulsion between hydrogen orbitals at their 60° vertices. We found that the addition of N–H or B–H pairs at 60° vertices mitigates these out-of-plane deformations. Surprisingly, the stability of triangular pores with N–H termination is significantly increased by the addition of B–H pairs, whereas the stability of triangular pores with B–H termination is decreased by the addition of N–H pairs, indicating a distinct evolution of the pore shape based on the initial edge termination. Furthermore, we observed that the combination of H and OH is more effective in stabilizing the edges of rhombic pores compared to using only H atoms, whereas the opposite is true for small triangular pores that cannot accommodate the ·OH radical. Our findings also demonstrate that rhombic pores have a high affinity for water absorption, much higher than that of triangular pores. This suggests that the type of pore can significantly alter the hydrophobicity of *h*-BN and influence water flow through the membrane. Additionally, we observed that water molecules tend to form hydrogen bonds with the N–H-terminated surface in rhombic pores, but not with the B–N-terminated surface, potentially leading to asymmetries in water flow through the pore area. Overall, our study provides valuable insights into the interaction between nanoporous *h*-BN membranes and water.

## Introduction

Water scarcity represents one of the greatest challenges faced by our societies because of changing climate patterns combined with growing water demand [1]. In the face of this problem, seawater desalination has gained significant attention. In recent decades, technological advances such as membrane technology and energy recovery equipment have led to a considerable reduction in the energy required to desalinate seawater [2–3]. The proposal to use membranes that exhibit superior selectivity and high water flux has been a major focus for desalination technology [4–6]. Computational methods have been employed to enhance the understanding of nanoscale desalination processes. In this context, the use of molecular dynamics and ab initio calculations allows for the study of the physics involved in nanostructured membrane materials designed to improve the desalination process.

In pursuit of greater efficiency in the desalination process, the scientific community has proposed membranes composed of various materials, including graphene [7–10], carbon nanotubes [11–14], molybdenum disulfide (MoS_2_) [15–18], and hexagonal boron nitride (*h*-BN) [19]. Among these materials, *h*-BN stands out because of its properties, which are similar to those of graphene. It is composed of alternating boron and nitrogen atoms arranged in a honeycomb-like crystalline structure, characterized by high thermal stability, low dielectric constant, and high mechanical strength [20–21]. Additionally, it possesses unique properties compared to graphene, such as a wide bandgap, electrical insulation, and chemical inertness.

Because of its remarkable mechanical properties and resistance to oxidation during the desalination process, *h*-BN can be used as a molecular sieve [22–25]. Theoretical studies using molecular dynamics simulations analyzed the impact of the partial charge on the *h*-BN membrane surface on water molecules and salt ion transport [26]. They noted that the Coulomb interaction between water molecules/ions and the channels influences the mobility of water/ions through the membrane. Consequently, the transport rates of both ions and water decrease when passing through the highly polarized membrane. Additionally, the salt rejection in the polarized channel increases in proportion to the channel length, accompanied by a reduction in both water and ion flow [27].

*h*-BN membranes can feature pores of various types and sizes, which play a crucial role in the flow of water. The difference in the electroaffinity of B and N atoms gives these pores edges with asymmetric charge distributions, resulting in fields that affect the passage of ions and polar molecules. However, the use of *h*-BN as a membrane for water desalination is still a relatively new area of research.

In this paper we propose an analysis using first-principles calculations to understand the stability of pores in monolayer *h*-BN and their interaction with water. The remainder of the paper is organized as follows: In section “Theoretical Methods”, we present the details of the calculations, in section “Results and Discussion”, results are shown and discussed, and section “Conclusion” brings the conclusions.

## Theoretical Methods

We performed calculations based on the density functional theory (DFT) as implemented in the SIESTA [28] code. For the calculations we have used the generalized gradient approximation (GGA), proposed by Perdew, Burke, and Ernzerhof (PBE) [29] as exchange–correlation functional. For the studies of water adsorption, we also use the BH van der Waals functional [30]. We make use of norm-conserving Troullier–Martins [31] pseudopotentials in the Kleinman–Bylander [32] factorized form. Also, we have used as basis set the standard double-ζ plus polarizations orbitals (DZP). The basis functions and the electron density were projected into a uniform real space grid defined using an energy cutoff of 200 Ry. The employed unit cells are periodic in the *x–y* directions; in the *z* direction, we have used vectors large enough to avoid the interaction between the periodic images.

In order to investigate the energetic stability of the *h*-BN nanopores, we calculated the formation energy according to Equation 1:


[1] $$E_{\text{f}}=E_{\text{total}}-n_{\text{N}}\mu_{\text{N}}-n_{\text{B}}\mu_{\text{B}}-n_{\text{H}}\mu_{\text{H}}-n_{\text{O}}\mu_{\text{O}},$$

where *E*_total_ and *n*_N_, *n*_B_, *n*_H_, and *n*_O_ are the total energy of the nanopore and the numbers of N, B, H, and O atoms for each studied system, respectively. μ_N_, μ_B_, μ_H_, μ_O_ are the chemical potentials of N, B, H, and O atoms, respectively. The atomic chemical potentials are obtained as follows:


[2] $$\mu_{\alpha }=\frac{\mu_{\text{source}}-n_{\text{H}}\frac{\mu_{{\text{H}}_{2}}}{2}}{n_{\alpha }},$$

where μ_α_ and *n*_α_ represent the chemical potential and the number of atoms of type α (α = N, B, H, O). The chemical potential of 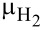 is derived from the total energy of the *H*_2_ molecule, while μ_source_ denotes the total energy of the systems comprising the chosen chemical reservoir (which may include molecules such as BH_3_, NH_3_, B_2_H_6_, H_2_O, and N_2_, or solid phases such as α-B and α-N) [33]. Here, *n*_H_ represents the number of hydrogen atoms in the pore. We presume chemical equilibrium between the sources of B and N atoms and the *h*-BN sheet. Consequently, the chemical potentials for B and N atoms are interrelated, that is, μ_B_ + μ_N_ = μ_BN_, where μ_BN_ signifies the chemical potential of the *h*-BN monolayer. Under this constraint, the formation energies of *h*-BN nanopores with stoichiometric ratios of boron and nitrogen atoms are independent of the individual choices of μ_B_ and μ_N_. This is because, for stoichiometric pores, Equation 1 can be reformulated as *E*_f_ = *E*_total_ − *n*_BN_μ_BN_ − *n*_H_μ_H_ − *n*_O_μ_O_. Conversely, nanopores may possess varying numbers of B and N atoms. Therefore, it becomes necessary to consider chemical environments favoring either μ_B_ (i.e., a B-rich environment) or μ_N_ (i.e., a N-rich environment).

**Figure 1:**
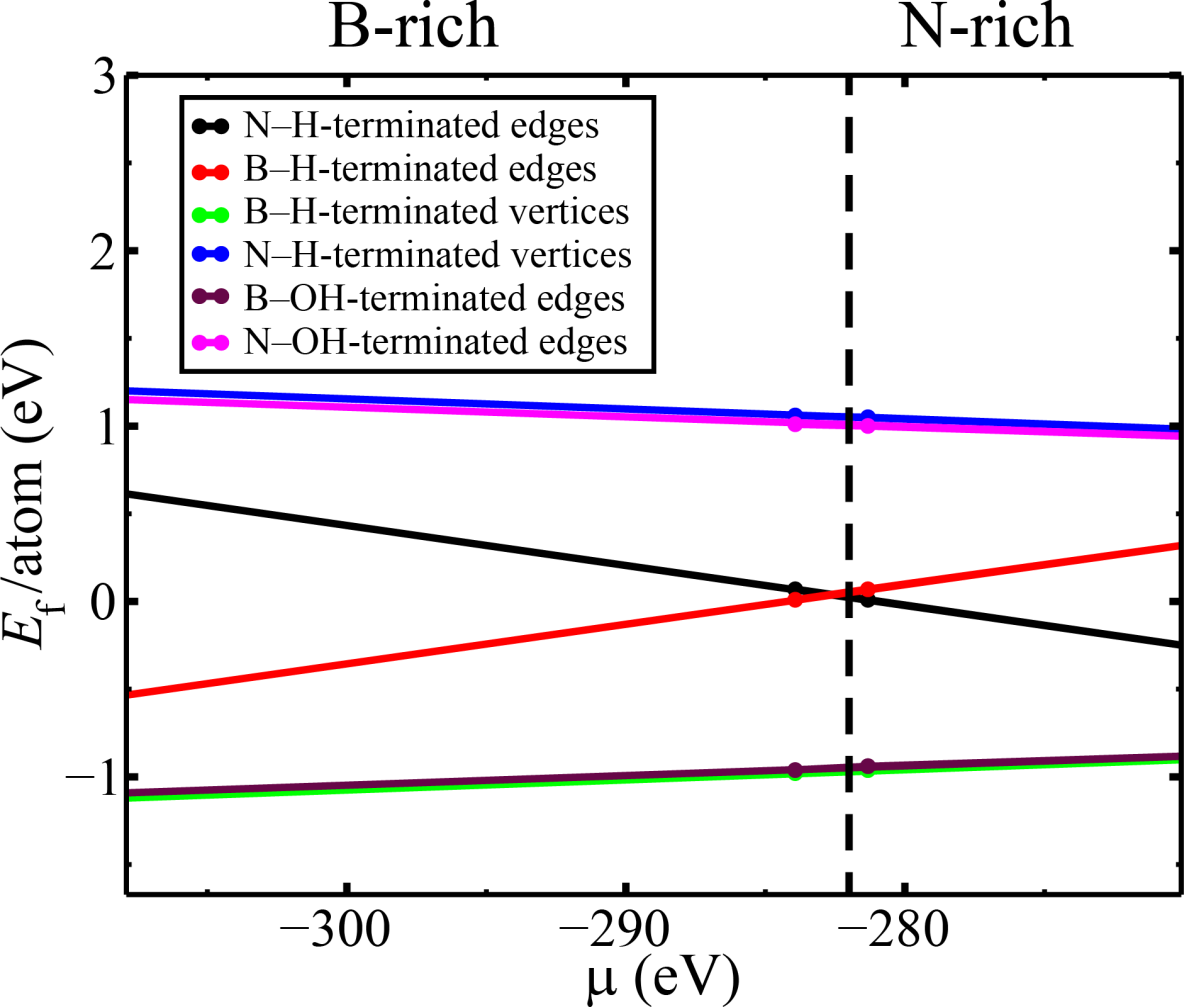
Formation energy, *E*_f_ per atom as a function of μ_N_, for triangular nanopores as shown below in Figure 3. From left to right, the circles mark the values of energy calculated at *T* = 0 K using the following sources of B or N atoms: BH_3_, α-B, NH_3_ and N_2_. BH_3_ and α-B determine μ_B_, while μ_N_ is obtained from the constraint μ_BN_ = μ_B_ + μ_N_.

To determine the chemical potential of nitrogen in N-rich environments, we utilize either the N_2_ molecule (whereby 
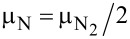
 and μ_B_ = μ_BN_ − μ_N_) or the NH_3_ molecule (in this case, 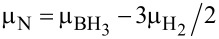 and μ_B_ = μ_BN_ − μ_N_). In boron-rich environments, the BH_3_ molecule is employed to determine μ_B_. As per Equation 2, we have 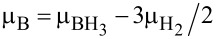. Our choice of BH_3_ and NH_3_/N_2_ as chemical reservoirs for, respectively, boron and nitrogen atoms corresponds to experimental conditions in which these molecules were utilized as reagents for synthesizing porous *h*-BN. Figure 1 demonstrates that over a wide range of μ_N_ values, the transition from a B-rich to a N-rich environment has a small effect on the formation energy of the two most stable porous *h*-BN structures. These structures remain significantly more energetically stable than the other triangular porous *h*-BN monolayers.

The adsorption energy, *E*_ads_, was calculated through Equation 3:


[3] $$E_{\text{ads}}=E_{\text{total}}-E_{\text{porous}}-E_{{\text{H}}_{2}\text{O}},$$

where *E*_total_ is the total energy of system, *E*_porous_ is the energy of the porous monolayer, and 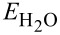 is the energy of an isolated water molecule.

## Results and Discussion

### Energetic stability of *h*-BN nanopore

**Figure 2:**
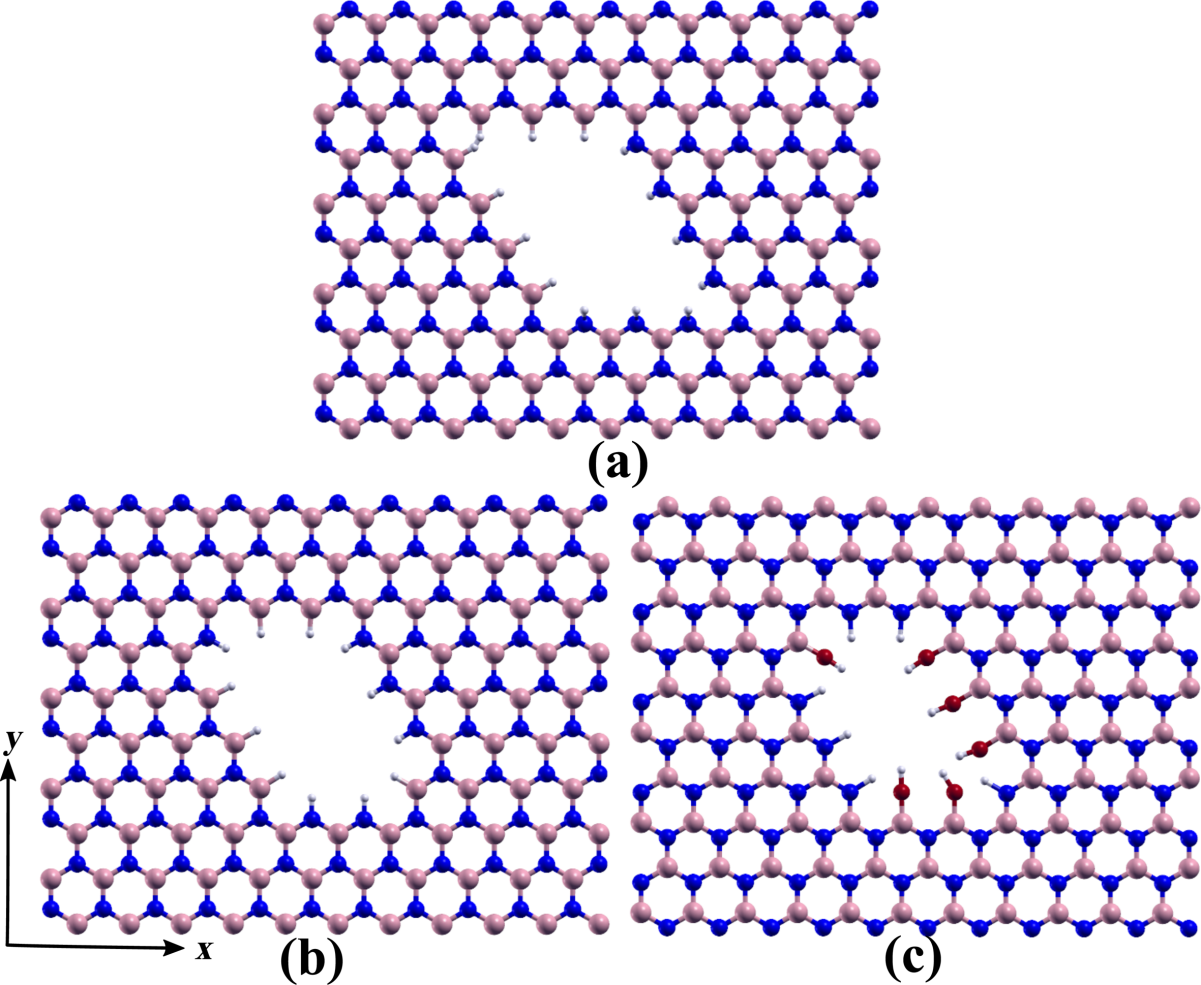
Rhombic nanopores in free-standing *h*-BN monolayers. (a) Rhombic pore with B–H and N–H edges. (b) Rhombic pore with B–H and N–H pairs at 60° vertices. (c) Rhombic pore with B–OH and N–H terminations.

**Figure 3:**
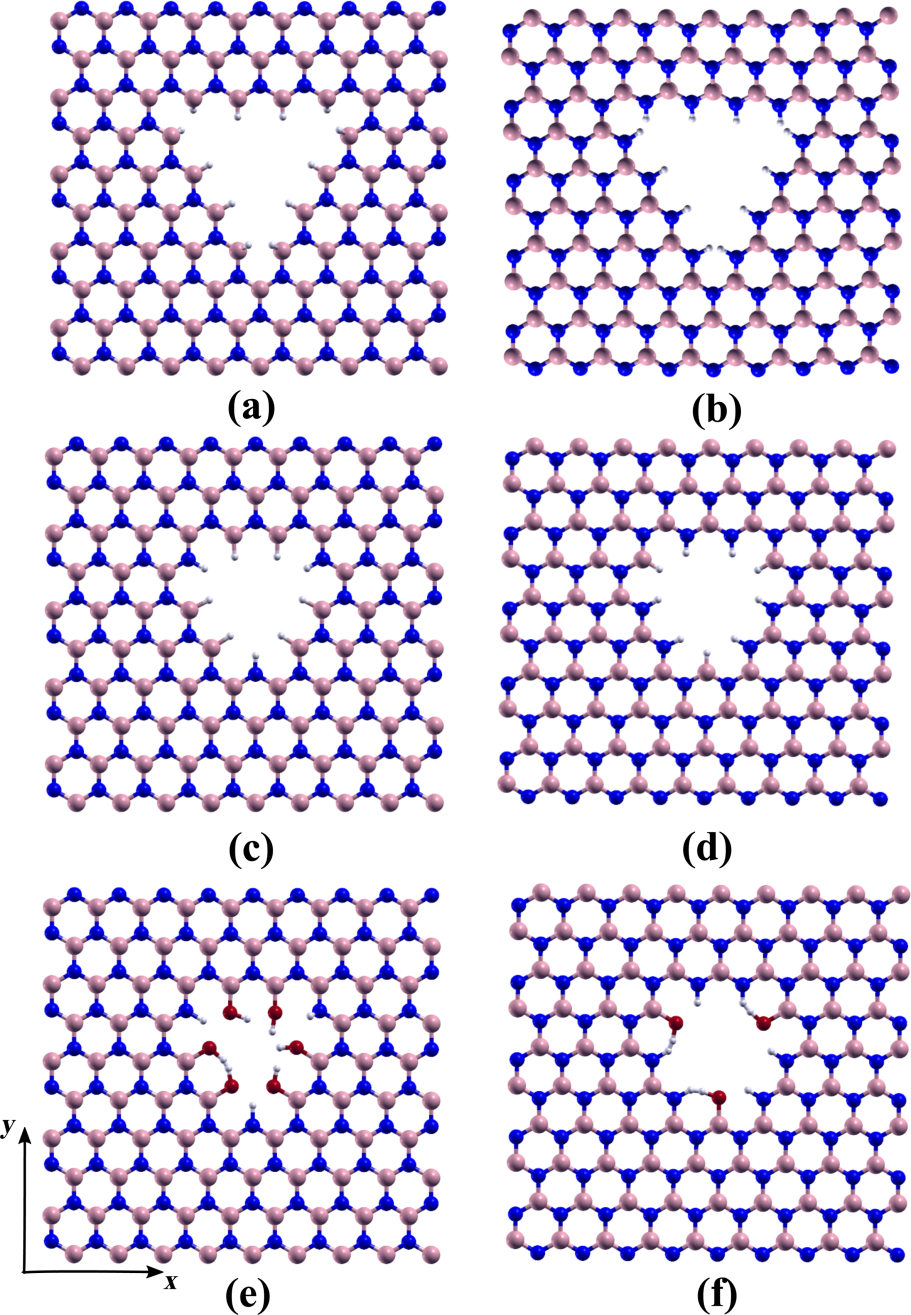
Triangular nanopores in free-standing *h*-BN monolayers. Triangular pores with (a) B–H and (b) N–H edges, triangular pores with (c) N–H and (d) B–H pairs at 60° vertices, and triangular pores with (e) B–OH and (f) N–H terminations.

Figure 2 and Figure 3 display the *h*-BN nanosheets with rhombic and triangular pores that were initially analyzed in this work. The rhombic and triangular pores were passivated with hydrogen atoms, as shown in Figure 2a,b and Figure 3a–d. After optimization, wrinkles were observed in both the rhombic and triangular porous structures, which were not observed in deposited pores [34]. These deformations arise from the repulsion between the hydrogen orbitals of atoms at the 60° vertices, as depicted in the top panels of Figure 2. The hydrogen atoms repel each other towards different sides of the *h*-BN sheet, inducing strain that propagates through the sheet, resulting in out-of-plane deformations.

This deformation can be mitigated by introducing either a boron or a nitrogen atom at each 60° vertex of the rhombus or triangle, as illustrated in Figure 2c and Figure 3e,f. Such vertex modifications can either improve or reduce the energetic stability of the pore, as we will elaborate further. Experimental evidence supports the occurrence of these kinds of vertex configurations [34]. Liu and colleagues [35] demonstrated that two-dimensional hexagonal boron nitride macromolecules exhibit enhanced stability when passivated with hydroxy (–OH) and secondary amino (–NH) groups. Inspired by this finding, we also explore such passivation at nanopore edges, as illustrated in Figure 2c and Figure 3e,f.

The findings presented in Table 1, consistent with previous literature [36–37], indicate that triangular pores with either B–H- or N–H-terminated edges (Figure 3a,b) can form because the calculated formation energy per atom is either negative or lower than *k*_B_*T* at room temperature (approximately 25 meV). Specifically, N–H and B–H terminations are likely to occur in N-rich and B-rich environments, respectively. Additionally, N–H/B–H-terminated stoichiometric parallelogram pores exhibit low values of formation energy per atom, suggesting their potential for experimental formation. Indeed, Pham et al. [34] observed both types of pores and noted that parallelogram- and hexagon-shaped defects with zigzag edges become prevalent at temperatures exceeding 700 °C, in contrast to the triangular defects typically observed at lower temperatures.

**Table 1:** Formation energy per atom of porous *h*-BN nanosheets with passivated edges in boron-rich and nitrogen-rich environments. The chemical potentials for nitrogen atoms were defined using either NH_3_, second column, or N_2_, third column. All values are in eV/atom.

Pores	NH_3_ and BH_3_		N_2_ and BH_3_	

triangular	B-rich	N-rich	B-rich	N-rich

N–H-terminated edges (Figure 3b)	0.070	0.010	0.650	−0.256
B–H-terminated edges (Figure 3a)	0.006	0.066	−0.575	0.332
B–H-terminated vertices (Figure 3d)	−0.980	−0.965	−1.125	−0.898
N–H-terminated vertices (Figure 3c)	1.061	1.046	1.207	0.980
B–OH-terminated edges (Figure 3e)	−0.956	−0.943	−1.100	−0.867
N-OH-terminated edges (Figure 3f))	1.003	1.018	1.161	0.938

rhombic	B-rich	N-rich	B-rich	N-rich

N–H/B–H-terminated edges (Figure 2a)	0.029	0.029	0.029	0.029
N–H/B–H-terminated vertices (Figure 2b)	0.025	0.025	0.025	0.025
N–H/B–OH-terminated vertices (Figure 2c)	−0.009	−0.009	−0.009	−0.009

The incorporation of B–H or B–OH moieties at the vertices of triangular pores results in a significant reduction in formation energies. The decrease in energy per atom ranges from −1.230 to −0.525 eV, depending on the chemical environment (B-rich or N-rich, defined by using N_2_ or NH_3_ reservoirs).

As previously discussed, the presence of additional B–H or B–OH moieties at the vertices alleviates the strain induced by repulsion between hydrogen orbitals at these locations, thereby reducing the elastic energy. However, the reduction in formation energy cannot be attributed solely to the reduction in elastic energy, which is of the order of 0.017 eV/atom. This suggests that chemical effects play a predominant role in the overall reduction of formation energy.

The reduction in energy per atom caused by the addition of B–H pairs in triangular pores is about 0.02 eV/atom smaller than that caused by the addition of B–OH moieties. This result differs from the findings of Liu and colleagues on *h*-BN macromolecules [35]. We attribute this difference to the heightened repulsion among atoms within the triangular pore, as B–OH moieties occupy more space than B–H pairs. In the case of *h*-BN macromolecules, as studied by Liu and colleagues, similar confinement effects do not exist, allowing B–OH terminations to enhance stability relative to B–H terminations. This is similar to the situation with larger pores, such as rhombic pores, where confinement effects are minimal or even negligible. For the rhombic pores, the one with B–OH-terminated vertices has the lowest formation energy.

It is noteworthy that the introduction of N–H pairs or N–OH moieties at the vertices of triangular pores significantly increases the formation energy, despite also relieving strain. This increase ranges from 0.586 to 1.236 eV, depending on the environment. Thus, the dynamics of pore formation for pores with B–H-terminated edges are expected to differ from those of N–H-terminated pores. Specifically, an additional B–H/B–OH group should migrate to the 60° vertices of pores to improve stability, whereas an additional N–H pair does not provide the same effect. In fact, Pham et al. observed that N-terminated pores are less stable in air than B-terminated ones [34].

### Water molecule in *h*-BN nanosheets

We studied the adsorption process of a water molecule on a porous *h*-BN nanosheet. Initially, we focused on the adsorption of the water molecule on a nanosheet with rhombic pores. The rhombic pore is particularly interesting because of its N–H and B–H terminations at opposite edges, resulting in differing charge distributions at these edges. Given that water is a polar molecule, it is expected to be influenced by the asymmetric charge distribution at the pore edges, which could affect its orientation and subsequently impact the dynamics of water flow through the pore.

**Figure 4:**
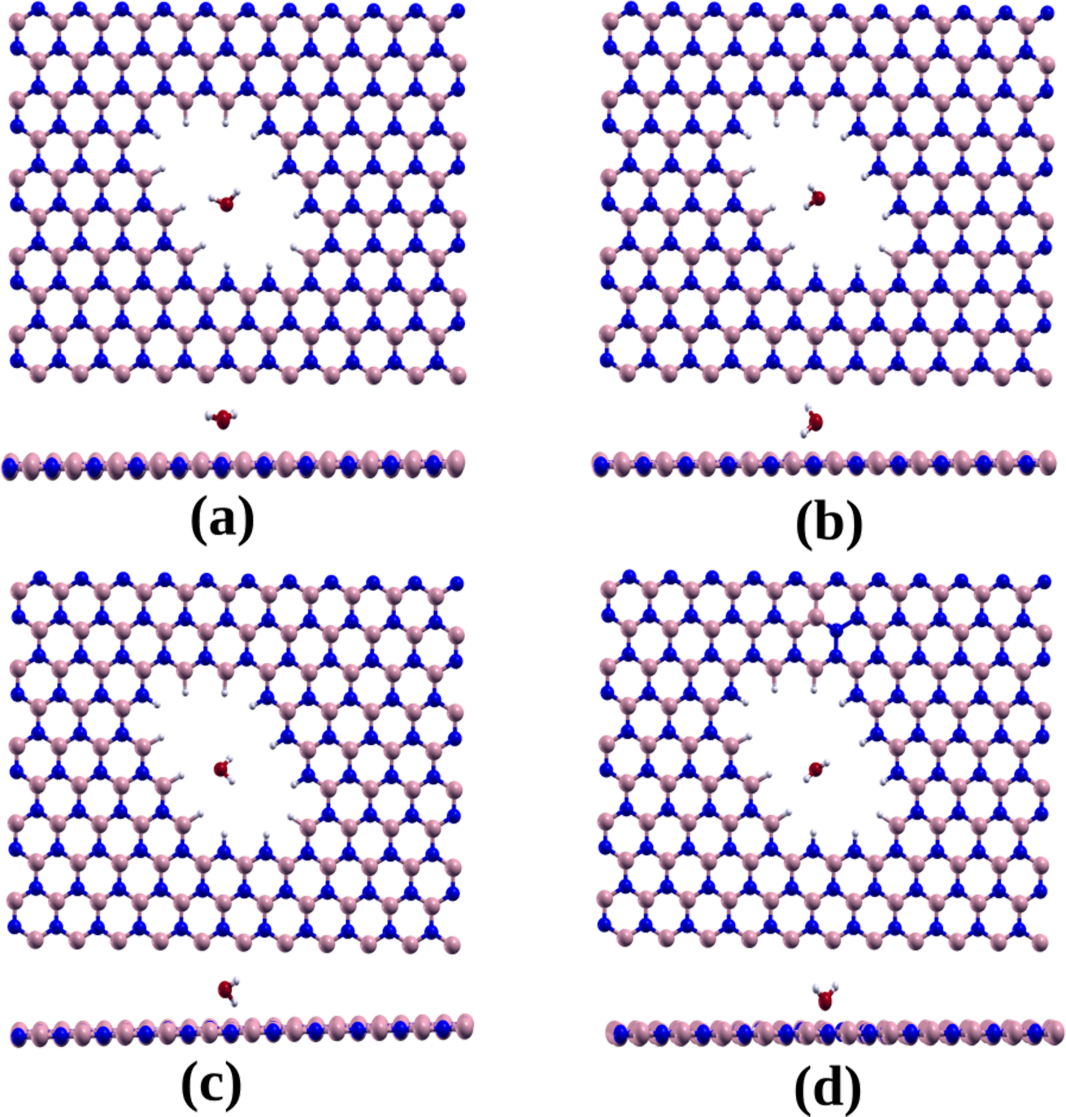
Diagram illustrating the various adsorption positions of the water molecule on *h*-BN nanosheets with rhombic pores, frontal view and view perpendicular to the adsorption plane. (a) Water molecule atoms parallel to the hBN monolayer; (b) water molecule atoms perpendicular to the *h*-BN monolayer, with the oxygen atom near the NH-terminated edge and the hydrogen atoms near the BH-terminated edges; (c) the plane containing the water molecule atoms perpendicular to the *h*-BN monolayer with the oxygen atom near the BH-terminated edge and the hydrogen atoms near the NH-terminated edges; and (d) the plane containing the water molecule atoms perpendicular to the *h*-BN monolayer, with the oxygen atom near the monolayer and hydrogen atoms directed away from the monolayer.

In our study, we examined four different initial orientations for water adsorption: (1) The plane containing the water molecule atoms is parallel to the hBN monolayer (Figure 4a); (2) the plane containing the water molecule atoms is perpendicular to the *h*-BN monolayer, with the oxygen atom near the NH-terminated edge and the hydrogen atoms near the BH-terminated edges (Figure 4b); (3) the plane containing the water molecule atoms is perpendicular to the *h*-BN monolayer, with the oxygen atom near the BH-terminated edge and the hydrogen atoms near the NH-terminated edges (Figure 4c); and (4) the plane containing the water molecule atoms is perpendicular to the *h*-BN monolayer, with the oxygen atom near the monolayer and hydrogen atoms directed away from the monolayer (Figure 4d).

For each orientation, the water molecule was initially positioned at the geometrical center of the pore, at distances of 1.0, 1.5, 2.0, and 2.5 Å above the *h*-BN monolayer, as shown in Figure 4. After geometry optimization, we observed that in three of the initial configurations, that is, orientations 1–3, the oxygen atom of the water molecule tends to form a dipole bond with the N–H-terminated edge, while one hydrogen atom of the water molecule forms a bond with the opposite B–H-terminated edge.

Table 2 presents the calculated adsorption energies for the different initial conditions. It can be observed that calculations using the BH functional result in significantly higher adsorption energies compared to those obtained using the PBE functional. For orientations 1–3, calculations with the van der Waals-corrected BH functional yield similar final geometries for each orientation (i.e., similar OH and H–H distances) regardless of the initial height. Correspondingly, the adsorption energy values are also similar, approximately −2.83, −2.93, and −2.92 eV for orientations 1, 2, and 3, respectively. In contrast, the optimized geometries and adsorption energies calculated using the PBE functional are more sensitive to the initial height of the water molecule. For the fourth initial configuration (Figure 4d), the optimization resulted in a final configuration where the oxygen atom is close to the B–H-terminated edge and the hydrogen atoms of the water molecule maintain similar distances from the N–H- and B–H-terminated edges.

**Table 2:** Adsorption energy values of a H_2_O molecule on a rhombic pore for different orientations and the minimum distance between the oxygen and hydrogen atoms of the water molecule to the hydrogen atoms at the pore edges.

Initial water	Adsorption energy(eV)	O–H distance (Å)	H–H distance (Å) )

molecule position	PBE (BH)	PBE (BH)	PBE (BH)

orientation 1 (*z* = 2.5 Å)	−0.78 (−2.82)	3.77 (2.07)	3.23 (3.36)
orientation 1 (*z* = 2.0 Å)	−0.97 (−2.83)	3.17 (2.07)	2.54 (3.16)
orientation 1 (*z* = 1.5 Å)	−1.03 (−2.82)	3.09 (2.12)	2.23 (3.12)
orientation 1 (*z* = 1.0 Å)	−**1.08** (−2.83)	2.83 (2.07)	2.43 (3.15)
orientation 2 (*z* = 2.5 Å)	−0.99 (−2.93)	3.33 (2.08)	2.19 (3.17)
orientation 2 (*z* = 2.0 Å)	−**1.21** (−2.93)	2.08 (2.05)	3.12 (3.21)
orientation 2 (*z* = 1.5 Å)	−1.07 (−2.91)	3.09 (2.05)	2.25 (3.20)
orientation 2 (*z* = 1.0 Å)	−1.20 (−2.93)	2.10 (2.05)	3.11 (3.15)
orientation 3 (*z* = 2.5 Å)	−1.12 (−2.91)	2.61 (2.08)	2.67 (3.27)
orientation 3 (*z* = 2.0 Å)	−1.07 (−2.90)	2.87 (2.03)	2.47 (3.26)
orientation 3 (*z* = 1.5 Å)	−**1.14** (−2.92)	2.37 (2.06)	2.79 (3.14)
orientation 3 (*z* = 1.0 Å)	−0.99 (−2.92)	3.01 (2.08)	2.81 (3.15)
orientation 4 (*z* = 2.5 Å)	−0.78 (−2.50)	3.33 (3.44)	3.02 (3.15)
orientation 4 (*z* = 2.0 Å)	−0.81 (−2.45)	3.07 (2.59)	2.73 (3.11)
orientation 4 (*z* = 1.5 Å)	−0.83 (−2.45)	2.92 (3.57)	2.52 (3.71)
orientation 4 (*z* = 1.0 Å)	−**0.84** (−2.87)	2.82 (2.59)	2.39 (3.11)

As presented in Table 2, orientation 4 exhibits higher adsorption energy values compared to the other three configurations. Overall, the adsorption energy ranges between −1.21 (−2.45) and −0.79 (−2.87) eV, indicating a strong affinity between the rhombic pore and the water molecule. Notably, this affinity is much higher than that observed in triangular pores, as we will discuss.

**Figure 5:**
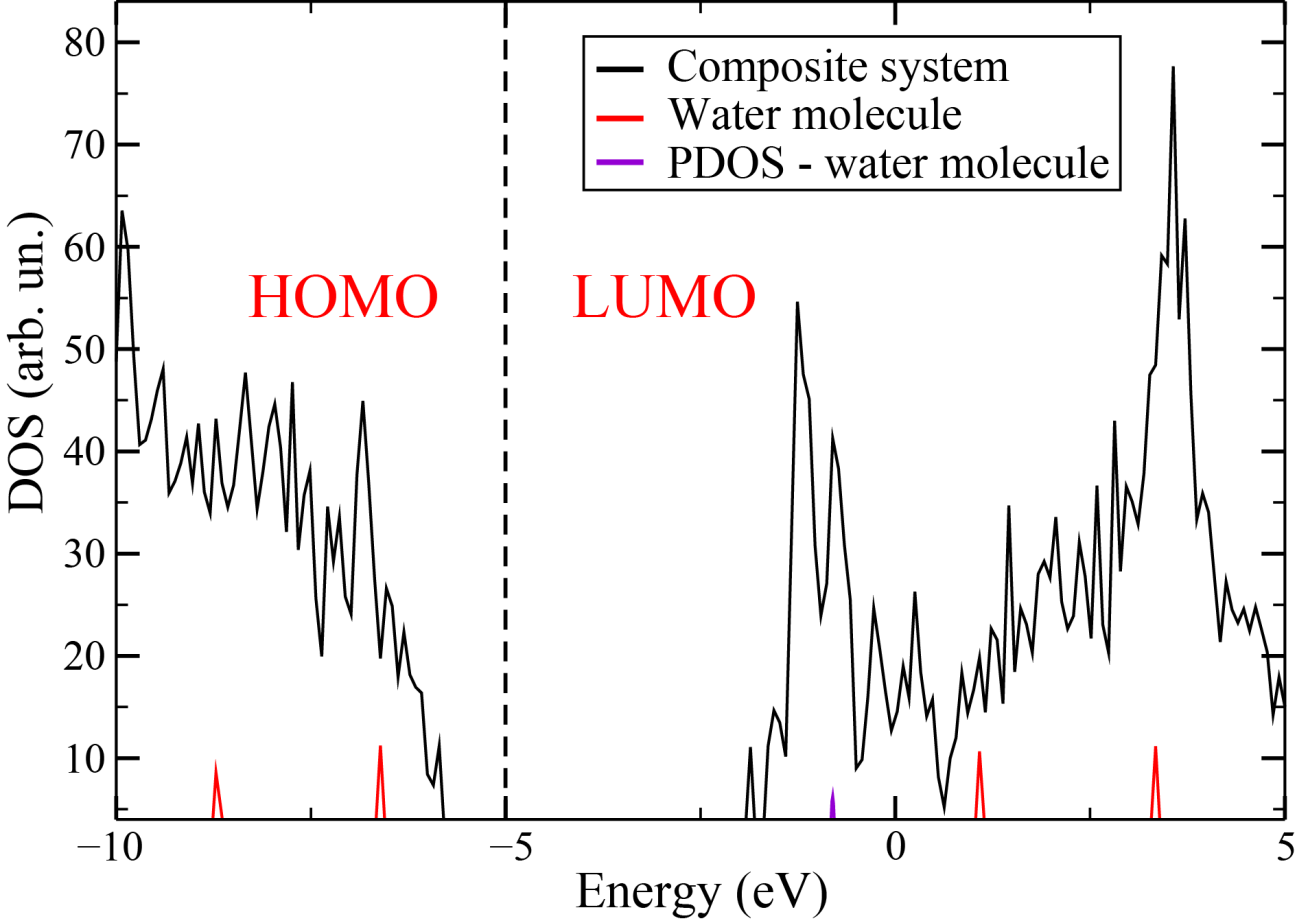
Density of states of a system composed of water and rhombic pore (black line), isolated water molecule (red line), and projected density (PDOS) for the water molecule in the composite system (violet line).

Figure 5 illustrates the density of states (DOS) for an isolated water molecule and the DOS of a system comprising a water molecule interacting with a rhombic pore. For the combined system, the graph also presents the DOS projected onto the atoms of the water molecule. It is evident that the positions and relative intensities of the water molecule peaks change significantly upon interaction with the porous *h*-BN. These changes align with the high binding energy observed between water and porous *h*-BN, despite Mulliken analysis indicating no significant charge transfer. The calculated O–-H bond lengths suggest the presence of hydrogen bonding, and a comparison of binding energies obtained using PBE and BH functionals indicates that van der Waals forces also play a substantial role in the interaction.

**Figure 6:**
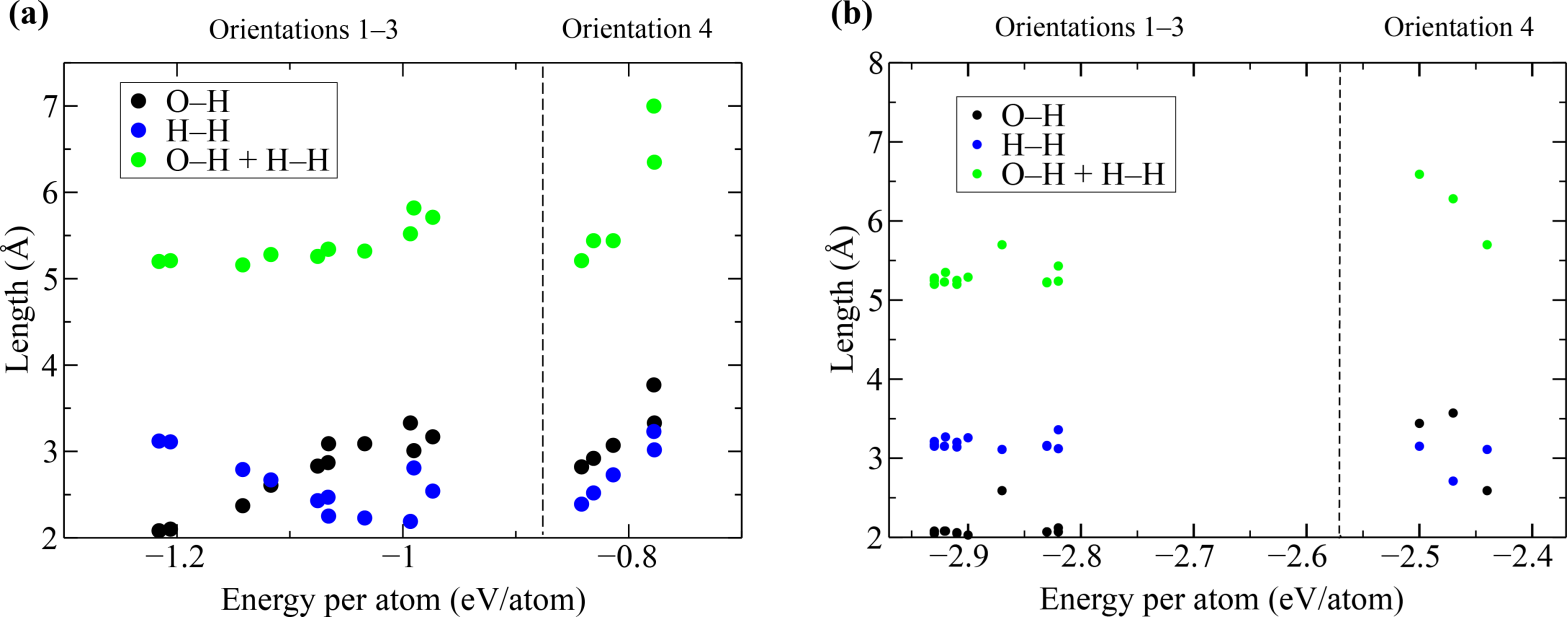
Distances between water molecule atoms and a hydrogen atom at N–H edges, orientations 1–3, and B–N edges, orientation 4, as a function of adsorption energy calculated using (a) the PBE functional and (b) the van der Waals-corrected BH functional.

Figure 6 illustrates the minimum distances between water atoms and pore edges as a function of adsorption energy, as also detailed in Table 2. For orientations 1–3, the sum of the O–H (oxygen of the water molecule and hydrogen of the N–H-terminated edge) and H–H (one hydrogen of the water molecule and the other of the B–H-terminated edge) distances (green circles) remains relatively constant for adsorption energies below −1 eV/atom, Figure 6a, and −2.9 eV/atom, Figure 6b. Notably, as the adsorption energy decreases, the O–H distance approaches approximately 3.1 Å, while the H–H distance approaches approximately 2.1 Å in a reciprocal manner in the PBE calculations. This pattern suggests that the oxygen atom of the water molecule tends to form a hydrogen bond with one of the hydrogen atoms at the N–H-terminated edge as the water molecule moves closer to the pore.

In orientation 4, the oxygen atom does not form a hydrogen bond with the B–H-terminated edge, as the O–H distance tends to be larger than the H–H distance. Therefore, we can speculate that the flow of water molecules through the rhombic pore is asymmetric regarding the pore area, with the water molecule spending more time near the N–H-terminated edge because of the stronger interaction with this edge.

We then analyzed the adsorption of a water molecule in triangular nanopores. Using the PBE functional, we found positive adsorption energy values (0.805 and 0.337 eV for B–N- and N–H-terminated triangles, respectively). Nevertheless, using the BH van der Waals-corrected functional, we found negative values, that is, −1.36 and −1.03 eV for B–N- and N–H-terminated triangles, respectively. The results indicate that the shape of the pore can significantly affect the hydrophilicity of the *h*-BN monolayer. Triangular pores render *h*-BN less hydrophilic than rhombic pores. This distinct behavior between triangular and rhombic pores suggests that pore shape can have a substantial impact on the flow of water through the material.

**Figure 7:**
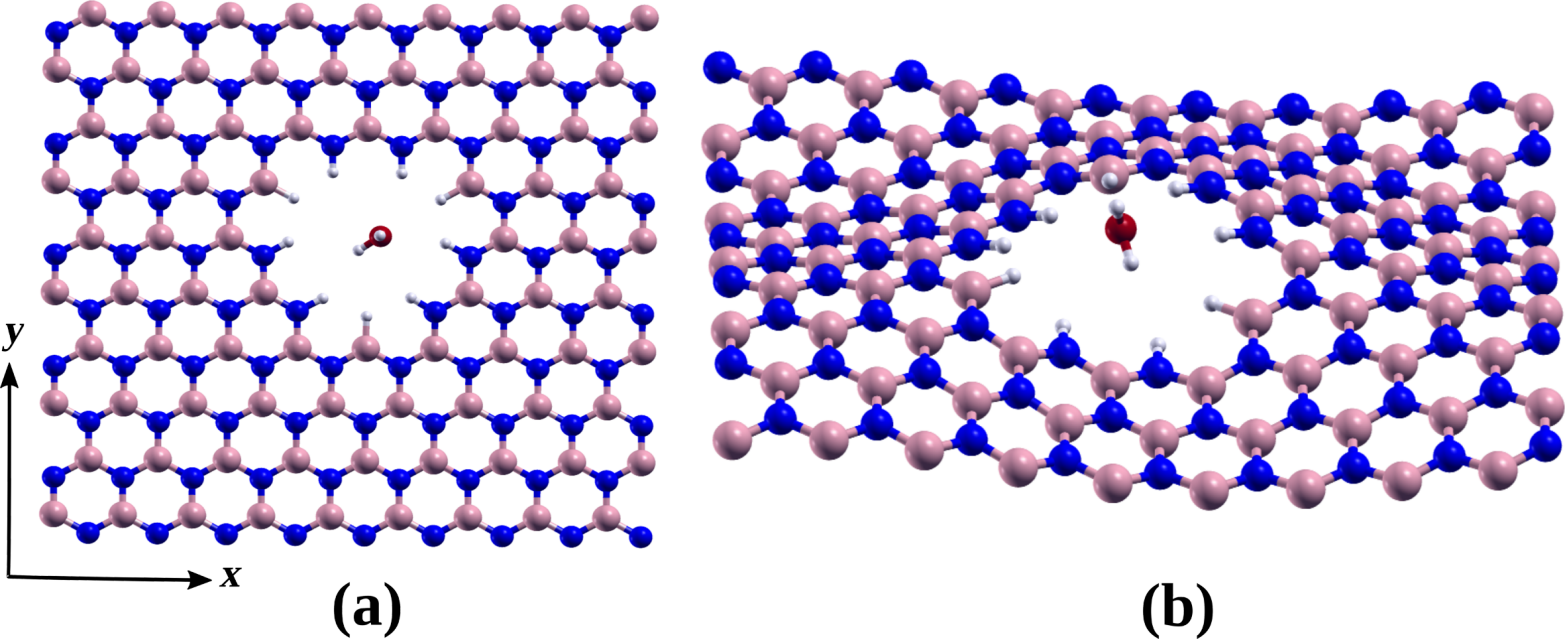
Illustration of the adsorption position of a water molecule on a *h*-BN nanosheet with triangular pores (a) before and (b) after optimization.

An intriguing aspect of the interaction between water and pores is the ability of a single water molecule to induce wrinkles in *h*-BN nanosheets with triangular pores, even when the pore is stabilized with B–H pairs at its vertices (see Figure 7). This deformation disappears upon removal of the water molecule. The observed deformation resembles that which occurs in triangular pores lacking additional B–H pairs at the vertices. Consequently, the strain energy due to deformation can be estimated to be approximately 3.05 or 0.017 eV per atom. This phenomenon aligns with the findings of Ferrari and colleagues [38] in their study of suspended graphene membranes interacting with water, where wrinkles significantly impact the mechanical response of the membranes when filled with water on a platform.

## Conclusion

Our investigation of pores in a free-standing hexagonal boron nitride monolayer sheds light on the intricate interactions between 2D nanoporous membranes and water. We observed that the addition of N–H or B–H pairs at 60° vertices mitigates wrinkles induced in triangular and rhombic pores. Surprisingly, the stability of N–H-terminated triangular pores significantly increases with the addition of B–H pairs, whereas the introduction of N–H pairs reduces the stability of B–H-terminated triangular pores. Additionally, we found that combining H and OH groups is more effective than using only H atoms to stabilize the edges of rhombic pores.

The distinct behavior of triangular and rhombic pores in their interaction with water suggests that the type of pore can alter the hydrophobicity of *h*-BN and influence the flow of water through it. Moreover, the formation of a hydrogen bond with the N–H-terminated surface in rhombic pores, which is absent in interactions with the B–N-terminated surface, may lead to asymmetries in water flow through the pore area.

## Data Availability

Data generated and analyzed during this study is available from the corresponding author upon reasonable request.
